# Reliability of Preoperative MRI Findings for Differentiating Spontaneous Spinal Subdural and Epidural Hematomas: A Multi-Institutional Retrospective Study of 27 Surgically Treated Cases

**DOI:** 10.3390/jcm15072602

**Published:** 2026-03-29

**Authors:** Shun Okuwaki, Hiroshi Takahashi, Katsuya Nagashima, Tomoyuki Asada, Takane Nakagawa, Takahiro Sunami, Yosuke Ogata, Kotaro Sakashita, Hisanori Gamada, Kousei Miura, Hiroshi Noguchi, Yosuke Takeuchi, Toru Funayama, Masao Koda, Masaki Tatsumura

**Affiliations:** 1 Department of Orthopedic Surgery, Institute of Medicine, University of Tsukuba, 1-1-1 Tennodai, Tsukuba 305-8575, Japan; hhtaka@tsukuba-seikei.jp (H.T.); takane.n@tsukuba-seikei.jp (T.N.); taka113sunami@tsukuba-seikei.jp (T.S.); yogata.tuk@tsukuba-seikei.jp (Y.O.); k.sakashita715@gmail.com (K.S.); hisanorigamada@tsukuba-seikei.jp (H.G.); kmiura@tsukuba-seikei.jp (K.M.); noguhiro0164@tsukuba-seikei.jp (H.N.); funatoru3@tsukuba-seikei.jp (T.F.); masaokod@tsukuba-seikei.jp (M.K.); 2 Department of Orthopaedic Surgery and Sports Medicine, Tsukuba University Hospital, Mito Clinical Education and Training Center, Mito Kyodo General Hospital, 3-2-7 Miyamachi, Mito 310-0015, Japan; katsu_n103@yahoo.co.jp (K.N.); yousuketa912@gmail.com (Y.T.); tatsumura@md.tsukuba.ac.jp (M.T.); 3 Hospital for Special Surgery, 535 East 70th Street, New York, NY 10021, USA; kjjk991@tsukuba-seikei.jp

**Keywords:** spinal subdural hematoma, spinal epidural hematoma, spinal hematoma, spinal cord injury

## Abstract

**Background/Objectives:** Spontaneous spinal subdural hematoma (SSSDH) is a rare and severe condition that causes rapid neurological decline. Spontaneous spinal epidural hematoma (SSEH) presents similarly but is more common, and surgical management differs because SSSDH requires an intradural approach. Few studies have assessed the reliability of magnetic resonance imaging (MRI) features used to distinguish SSSDH from SSEH in patients requiring surgery. **Methods:** We retrospectively reviewed 27 patients who underwent surgical evacuation of spinal hematomas at two institutions (2015–2025). Definitive hematoma location was determined intraoperatively. Four MRI features—shape (crescentic vs. biconvex), location (ventral vs. dorsal), craniocaudal length (<5 vs. ≥5 segments), and spinal region—were independently evaluated by two reviewers. Inter- and intra-rater reliability was assessed using agreement rate and Cohen’s kappa (κ) with 95% confidence intervals (95% CIs). **Results:** Among 27 cases, three (11.1%) were SSSDH and 24 were SSEH. Hematoma location, length, and spinal region demonstrated perfect inter- and intra-rater agreement (κ = 1.00). For hematoma shape, intra-rater agreement was good (96.2%, κ = 0.84; 95% CI 0.52–1.00), whereas inter-rater agreement was poor to fair (84.6%, κ = 0.26; 95% CI −0.25–0.77). Notably, two of the three SSSDHs demonstrated a biconvex configuration, and 83.3% of SSEHs also exhibited a biconvex morphology. **Conclusions:** MRI features such as hematoma location, extent, and spinal level were highly reproducible, whereas hematoma shape showed limited reliability. Although ventral hematomas most strongly suggest SSSDH, atypical SSEH presentations occur. When dorsal exposure reveals no epidural hematoma, intradural exploration should be promptly considered.

## 1. Introduction

Spontaneous spinal subdural hematoma (SSSDH) is an extremely rare condition, often presenting with severe and rapid neurological deterioration that may result in irreversible deficits [1,2]. Long-term morbidity has been reported in up to 28% of cases, with mortality reaching up to 1.3% [2]. It is commonly linked to anticoagulant therapy or dural puncture, although idiopathic cases also occur [3]. Clinical presentation typically begins with an acute onset of local or radicular pain, followed by motor, sensory, and autonomic deficits. The pathogenesis of SSSDH remains unclear, but proposed mechanisms include rupture of radiculomedullary vessels traversing the subarachnoid space and secondary extension of subarachnoid hemorrhage into the subdural space [4]. Spontaneous spinal epidural hematoma (SSEH) is another rare but critical spinal emergency with a similar presentation [5]. The reported incidence of spontaneous spinal hematomas is extremely low, estimated at approximately 0.1 per 100,000 individuals per year [6]. SSEH is thought to result primarily from rupture of the valveless internal vertebral venous plexus in the epidural space due to sudden increases in venous pressure [7]. Both conditions require urgent surgical decompression by hematoma evacuation when severe motor deficits are present, but surgical strategies differ: SSSDH requires intradural exposure and hemostasis. Accurate preoperative differentiation is therefore essential.

Magnetic resonance imaging (MRI) is the gold standard for evaluating spinal hematoma [1]. Classical imaging features of SSSDHs include a crescent shape and a ventral location, extending across more than five intervertebral segments and the thoracolumbar region [8,9,10,11,12]. However, atypical findings are frequently reported, and hematoma morphology variability often precludes definitive distinction between subdural and epidural lesions. Although descriptors are commonly used to distinguish spinal hematomas from intracranial hematomas [13], no detailed studies have examined whether these morphological descriptors can be validly and reliably applied to spinal hematomas.

Prior studies investigating the radiological characteristics of spinal hematomas were generally small, without evaluating inter- and intra-rater reliability. To address this gap, we conducted a multi-institutional retrospective case series of surgically treated intraspinal hematomas to evaluate the reproducibility and diagnostic reliability of preoperative MRI features used to distinguish SSSDH from SSEH.

## 2. Materials and Methods

### 2.1. Patient Inclusion and Ethical Declaration

This retrospective study included patients diagnosed with SSSDH or SSEH of the cervical, thoracic, or lumbar spine who underwent hematoma evacuation at two institutions from April 2015 to March 2025. Only cases in which the hematoma location (subdural or epidural) was definitively identified intraoperatively by direct visualization performed by board-certified spinal surgeons were included. Exclusion criteria were: (1) non-surgical treatment; (2) no preoperative MRI; (3) hematomas secondary to malignancy; (4) trauma; or (5) iatrogenic causes (lumbar puncture, spinal anesthesia, or epidural catheterization), because these hematomas have a clearly identifiable procedural etiology and different clinical contexts compared with spontaneous hematomas. Surgery was determined at the attending surgeon’s discretion, even in mild cases, after discussing risks with patients. A total of 27 patients were included: 24 with SSEH and 3 with SSSDH. Demographic and clinical data (age, sex, imaging features, Frankel classification at presentation, and final follow-up) were collected from medical records.

The requirement for written informed consent was waived by the institutional review boards of both institutions, and an opt-out approach was implemented via the institutions’ websites, in accordance with the Declaration of Helsinki.

### 2.2. MRI Assessment

Preoperative MRI was assessed for four imaging features [8,9,10,11,12]: (1) hematoma shape (crescentic vs. biconvex), (2) location relative to the spinal cord (ventral vs. dorsal), (3) craniocaudal length (<5 vs. ≥5 intervertebral segments), and (4) spinal region (cervical, cervico-thoracic, thoracic, thoraco-lumbar, and lumbar). MRI sequences typically included sagittal and axial T1- and T2-weighted images.

Two board-certified spine surgeons independently reviewed the images, blinded to intraoperative findings. One rater repeated all ratings with a washout period of three months without access to prior assessment for intra-rater analysis. For inter-rater reliability, analyses from both raters were compared.

### 2.3. Statistical Analysis

Descriptive statistics summarized demographic and clinical variables. Comparative analyses between SSSDH and SSEH were not performed due to the rarity of SSSDH (n = 3). Intra- and inter-rater reliability was assessed using percent agreement and Cohen’s kappa coefficients (κ) with 95% confidence intervals (95% CIs). Statistical analyses were performed using R (ver. 4.4.3, R Core Team (2024), Vienna, Austria).

## 3. Results

Among 27 patients (mean age 64.7 ± 15.9 years; range 31–92), 18 were male and 9 were female. SSEH was mainly dorsal (95.8%), whereas two of the three SSSDH cases were ventral (Table 1). The cervical spine was the most frequently affected region in SSEH. Two of the three SSSDHs demonstrated a biconvex configuration, and 83.3% of SSEHs also exhibited a biconvex morphology.

Among the three SSSDH cases, two occurred in elderly male patients (71 and 83 years old) who were on chronic hemodialysis and receiving apixaban, while the remaining case involved a 31-year-old man with no history of anticoagulation therapy. Preoperative Frankel classification grades were A (n = 4), B (n = 3), C (n = 8), D (n = 9), and E (n = 3). The final follow-up period was 21 days to 9 years (Table 2).

Inter-rater and intra-rater agreement of hematoma location, length, and spinal regions achieved 100% agreement (κ = 1.00). For shape, intra-rater percent agreement was 96.2% (κ = 0.84; CI, 0.52–1.00), indicating good agreement. Inter-rater percent agreement was 84.6% (κ = 0.26; 95% CI, −0.25–0.77), reflecting poor–fair agreement. The discrepancy between high observed agreement and a lower κ reflects the influence of an imbalanced category distribution (κ paradox) in a small sample.

### 3.1. Case Presentation 1 SSSDH

**Figure 1:**
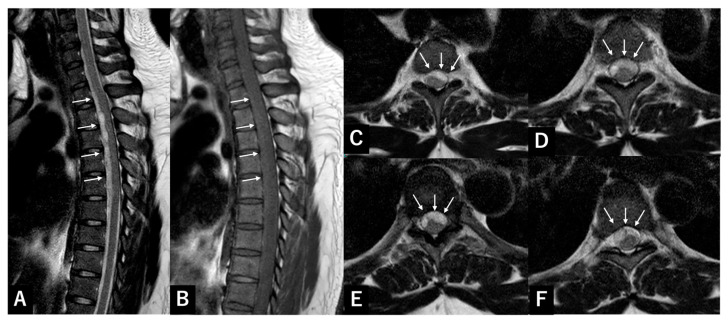
Representative case of SSSDH. (**A**) T2-weighted sagittal MRI of the cervicothoracic spine showing a ventral spinal hematoma (arrow) at T1–T6; (**B**) T1-weighted sagittal plane; (**C**) T2-weighted axial plane at T2/3; (**D**) T3/4; (**E**) T4/5; (**F**) T5/6. SSSDH: spontaneous spinal subdural hematoma; MRI: magnetic resonance imaging.

A 31-year-old man with no trauma or anticoagulant history developed sudden back pain and bilateral lower extremity paralysis. Manual muscle testing (MMT) was 0/5 with sensory loss below the xiphoid, bilateral proprioception loss, areflexia, and absence of anal tone. MRI revealed a ventral spinal hematoma at T1–T6 (Figure 1). The lesion was biconvex, hyperintense on T2- and hypointense on T1-weighted images.

Laboratory tests showed coagulopathy: aPTT 30.0 s, platelets 24,800/mm^3^, hemoglobin 17.3 g/dL, INR 0.93, and PT 11.7 s.

T2–T6 laminectomy was performed five hours after symptom onset. Although SSEH was initially suspected, no hematoma was found in the epidural space. The lesion was then identified as a subdural hematoma, and durotomy was performed. Ventral clots were successfully removed. Motor and sensory deficits persisted initially but gradually improved over time. At the six-month follow-up, Brown-Séquard syndrome remained, with residual paresis and hypoesthesia below T8.

### 3.2. Case Presentation 2 SSEH

**Figure 2:**
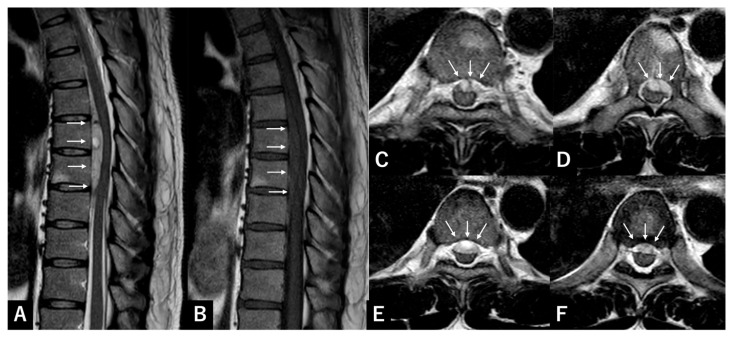
Representative case of SSEH. (**A**) T2-weighted sagittal MRI of the thoracic spine showing a ventral spinal hematoma (arrow) at T6–T8; (**B**) T1-weighted sagittal plane; (**C**) T2-weighted axial plane at T5/6; (**D**) T6; (**E**) T6/7; (**F**) T7/8. SSEH: spontaneous spinal epidural hematoma; MRI: magnetic resonance imaging.

A 34-year-old man developed acute back pain during exercise, followed by weakness in the left lower extremity. The patient had no history of trauma or anticoagulant use. MMT was 1/5, with hypoesthesia below the xiphoid process, bilateral proprioception loss, and absent anal tone. MRI showed a ventral T5–T8 crescent-shaped hematoma, hyperintense on T2- and hypointense on T1-weighted images (Figure 2).

Laboratory tests showed coagulopathy: aPTT 31.6 s, platelets 32,100/mm^3^, hemoglobin 16.1 g/dL, INR 1.00, and PT 12.3 s.

T5–T7 laminectomy was performed successfully five hours after symptom onset. Although SSSDH was initially suspected, durotomy revealed no hematoma within the subdural space. Instead, the hematoma was identified on the ventral aspect of the spinal cord within the epidural space. Thirty days postoperatively, the patient regained full motor and sensory function except for minimal lower limb spasticity, without bowel or bladder dysfunction.

No significant vascular abnormalities or tumoral lesions were observed in any of the cases.

## 4. Discussion

This study is, to our knowledge, the first multi-institutional case series to systematically evaluate the reliability of preoperative MRI features in distinguishing SSSDH from SSEH. A notable strength of our series is that the final diagnosis in all cases was confirmed intraoperatively, allowing for definitive compartmental assignment and eliminating diagnostic uncertainty that can occur in conservatively managed cases. We found that hematoma length and location showed high reliability, while hematoma shape showed only poor to fair agreement. When a hematoma is located ventrally, the likelihood of SSSDH is relatively high, as 66.7% of SSSDH cases in our series were ventral. However, because ventral hematomas were also observed in 4.2% of SSEH cases, an epidural origin should still be considered. These findings highlight the challenge of making a confident preoperative diagnosis, even for experienced spine surgeons. The occurrence in young patients without identifiable risk factors further underscores the unpredictable and heterogeneous nature of these lesions, emphasizing the need for caution when interpreting imaging features and planning surgery, particularly when an intradural approach is required.

The main clinical difficulty is differentiating SSSDH from SSEH based on MRI. Previous studies were mostly small case series describing potential features without assessing reproducibility [8,9,10,11,12]. Early reports suggested that SSSDHs often extend ventrally with a crescentic configuration and preservation of epidural fat, whereas SSEHs are more frequently located on the dorsal aspect and biconvex in contour [14,15,16,17]. Later pictorial reviews and radiological studies introduced additional signs such as the “inverted Mercedes-Benz sign” for SSSDH [12,18,19,20]. Although valuable in shaping diagnostic expectations, these criteria were anecdotal and descriptive, and never systematically validated for the inter- or intra-rater reliability. Consistent with prior reports, our findings suggest that descriptive signs alone are insufficient in emergency settings, especially when hematomas are hyperacute, compartmentalization is unclear, or image quality and mass effect are suboptimal [10].

Among the evaluated MRI features, hematoma location showed perfect inter-rater agreement (100%) and provided the clearest distinction between SSSDH and SSEH. In our cohort, 23 of 24 SSEHs (95.8%) were dorsal, while two of the three SSSDHs were ventral. The dorsal predominance of SSEH is consistent with current pathophysiology: rupture of fragile posterior epidural veins, often triggered by abrupt intrathoracic or intra-abdominal pressure changes, together with the wider posterior epidural space, promotes dorsal blood accumulation [21,22]. Notably, one SSEH was ventral, a location classically associated with a subdural origin. Previous reports indicate that ventral SSEH accounts for less than 10% of SSEH cases [23]. Although uncommon, ventral SSEH has been reported and may reflect alternative bleeding sources, including arterial rupture or variations in venous anatomy, underscoring that location alone cannot provide a definitive compartmental diagnosis [24]. Nevertheless, this variation stems from the pathophysiological variability, suggesting the relative reliability of location as a diagnostic feature in our results. Importantly, as demonstrated in our representative cases, even when hematomas are located ventrally, distinguishing whether they are subdural or epidural remains extremely difficult preoperatively. When hematomas appear ventral to the spinal cord, we recommend quick epidural exploration, and if no clear compressive lesion is found, prompt durotomy should be considered. This strategy may minimize delays and avoid further neurological deterioration.

In contrast, hematoma shape demonstrated poor to fair inter-rater reliability, reflecting the subjective nature of hematoma morphology. Even within our SSSDH cases, the lesion morphology differed from biconvex to crescentic, and not all lesions extended over five or more intervertebral segments, as traditionally described. Such inconsistencies complicate preoperative identification. As the hematoma evolves, assessment of the hematoma shape is time-dependent. It is sensitive to imaging plane, partial volume effects, and severe cord compression, which can flatten or distort contours in either compartment due to the mass effect, particularly in patients with severe motor deficits. Moreover, a distinct subdural boundary on T2-weighted images was frequently absent, further limiting confidence in the compartment assignment. These factors suggest that shape should be considered an ancillary, not definitive, sign. Similarly, while craniocaudal hematoma length showed perfect inter- and intra-rater agreement, its diagnostic utility was limited. Length, like shape, is influenced by imaging parameters and hematoma evolution. Together, these results show the limitations of relying solely on descriptive imaging features and the importance of cautious interpretation in clinical decision-making.

This study has several limitations. First, the retrospective design and small number of SSSDH cases limit generalizability. Second, only T1- and T2-weighted MRI sequences were assessed, and the reliability of other modalities could not be evaluated in emergent preoperative settings. Third, postoperative outcomes were not analyzed, limiting conclusions about prognosis. Fourth, the focus on surgical cases introduces selection bias, as conservatively treated patients were not included. This bias could either over- or underestimate the diagnostic performance of MRI features. Future studies should refine radiological criteria for SSSDH, using high-resolution T2-weighted sequences, contrast-enhanced imaging, or diffusion tensor imaging. Development of radiological scoring systems that incorporate the location, configuration, and extent may aid preoperative stratification. Large multicenter registries are also needed to better define idiopathic SSSDH profiles and identify new risk factors.

## 5. Conclusions

We found that hematoma location was the most reliable feature, while hematoma shape showed only fair inter-rater agreement, reflecting the subjectivity of morphological interpretation. These findings highlight the limitations of relying on classical imaging signs alone and emphasize the need to integrate imaging with clinical and intraoperative findings, especially when surgical planning requires intradural exposure.

## Figures and Tables

**Table 1:** Patient demographic data.

	SSSDH, n = 3	SSEH, n = 24
Age (mean ± SD, range)	64.7 ± 15.9, 31–92	
Sex, Male:Female	3:0	15:9
Shape (%)		
biconvex	2 (66.7)	20 (83.3)
crescent	1 (33.3)	4 (16.7)
Location (%)		
ventral	2 (66.7)	1 (4.2)
dorsal	1 (33.3)	23 (95.8)
Length (%)		
≥5	2 (66.7)	14 (58.3)
<5	1 (33.3)	10 (41.7)
Region (%)		
cervical	0	15 (62.5)
cervico-thoracic	1 (33.3)	4 (16.7)
thoracic	2 (66.7)	2 (8.3)
thoraco-lumbar	0	3 (12.5)
lumbar	0	0

SSSDH, spontaneous spinal subdural hematoma; SSEH, spontaneous spinal epidural hematoma; SD, standard deviation.

**Table 2:** Summary of imaging and clinical features of spinal hematomas.

Case	Diagnosis	Age	Sex	Level	Shape	Location	Anticoagulant Therapy	Preoperative Frankel Classification	Follow-Up Period	Frankel Classification at Final Follow-Up
1	SSSDH	31	M	T1–6	biconvex	ventral	none	A	1 month	C
2	SSSDH	72	M	C7–T2	biconvex	ventral	hemodialysis	C	3 months	D
3	SSSDH	83	M	T6–10	crescent	dorsal	apixaban	B	2 years	C
4	SSEH	70	M	C3–7	crescent	dorsal	aspirin, cilostazol	C	3 years	E
5	SSEH	51	M	C4–6	biconvex	dorsal	clopidogrel	D	9 years	E
6	SSEH	80	F	T10–L1	biconvex	dorsal	none	C	5 years	D
7	SSEH	70	M	C2–T1	biconvex	dorsal	aspirin	D	9 months	E
8	SSEH	68	F	C3–6	crescent	dorsal	none	D	5 years	E
9	SSEH	92	F	C3–6	biconvex	dorsal	none	A	42 days	C
10	SSEH	74	F	C3–4	biconvex	dorsal	cilostazol	D	1 year	E
11	SSEH	59	M	C2–7	crescent	dorsal	none	E	1 year	E
12	SSEH	75	F	C4–5	biconvex	dorsal	none	D	2 years	E
13	SSEH	71	M	C2–6	biconvex	dorsal	none	D	5 years	E
14	SSEH	46	M	C2–5	biconvex	dorsal	none	D	6 years	E
15	SSEH	52	F	T7–L1	biconvex	dorsal	none	A	4 years	D
16	SSEH	49	M	C2–3	biconvex	dorsal	none	E	5 years	E
17	SSEH	83	F	C3–T1	biconvex	dorsal	aspirin, cilostazol	C	5 years	E
18	SSEH	42	M	C2–5	biconvex	dorsal	none	E	4 years	E
19	SSEH	78	M	C2–5	biconvex	dorsal	rivaroxaban, cilostazol	C	33 days	C
20	SSEH	60	F	C2–6	biconvex	dorsal	none	D	4 years	E
21	SSEH	48	M	T9–12	biconvex	dorsal	none	B	3 years	E
22	SSEH	62	M	T12–L1	biconvex	dorsal	none	D	1 year	E
23	SSEH	57	M	C3–5	biconvex	dorsal	none	C	6 months	E
24	SSEH	81	M	C2–T2	biconvex	dorsal	none	B	21 days	D
25	SSEH	34	M	T5–8	biconvex	ventral	none	C	1 year	E
26	SSEH	85	F	C2–6	biconvex	dorsal	none	A	1 month	C
27	SSEH	73	M	C6–T6	biconvex	dorsal	none	C	3 years	E

SSSDH, spontaneous spinal subdural hematoma; SSEH, spontaneous spinal epidural hematoma.

## Data Availability

The data presented in this study are available on request from the corresponding author.

## Abbreviations

The following abbreviations are used in this manuscript:

CI	Confidence Interval
INR	International Normalized Ratio
MRI	Magnetic Resonance Imaging
MMT	Manual Muscle Testing
PT	Prothrombin Time
SSSDH	Spontaneous Spinal Subdural Hematoma
SSEH	Spontaneous Spinal Epidural Hematoma
